# The relationships between hoarding-related behaviours and disordered eating in a non-clinical adult sample: an exploratory study

**DOI:** 10.1186/s40359-025-03908-1

**Published:** 2026-03-27

**Authors:** Katri Cornelissen, Jiri Gumancik, Jane Scott, Nick Neave

**Affiliations:** 1 Hoarding Research Group, Northumbria University, Newcastle upon Tyne, NE1 8ST UK; 2 Posessions and Hoarding Collective, School of Psychology and Sport Science, Anglia Ruskin University, Cambridge, CB1 1PT UK

**Keywords:** Hoarding, Eating disorders, Perfectionism, Mood, Non-clinical sample

## Abstract

The prevalence of disordered eating is reportedly high in people with hoarding behaviours, and both eating disorders (ED) and hoarding disorder (HD) show strong associations with anxiety/depression. There is also some evidence that both may be underpinned by aspects of perfectionism. In a non-clinical sample of 252 adults (187 females) we explored possible relationships between disordered eating and hoarding severity, and assessed the relationships between hoarding, eating disorders and perfectionism. After controlling for gender and mood (anxiety and depression) we found significant positive correlations between hoarding symptoms (especially ‘acquisition’) and one aspect of disordered eating (bulimic severity). We did not find an association between hoarding behaviours and perfectionism as a whole, but did find a significant negative association between hoarding and ‘organization’, which is one aspect of perfectionism. The predicted association between ED and perfectionism was found at all levels of analysis. We propose that hoarding-related behaviour (especially excessive acquisition) may be associated most strongly with binge-eating and this should be explored further in future research.

## Introduction

 According to the American Psychiatric Association [1], Hoarding Disorder (HD) is characterised by (a) the excessive accumulation of items, (b) severe difficulty in discarding those items, (c) leading to extreme cluttering of living spaces, which prevents their intended use, and (d) clinically significant distress or impairment in normal functioning. HD is a chronic condition, emerging in late adolescence but becoming more problematic with age, as it clearly takes time to accumulate possessions to the point of severe clutter, e.g. [2]. The prevalence of HD is around 2.5%, making it more common than disorders such as schizophrenia and OCD [3], although some researchers have reported a higher incidence of up to 6% [4]. While it may present as an eccentric but harmless lifestyle choice, hoarding is becoming increasingly associated with poor social and economic outcomes, marginalisation, deprivation, increasing health problems and accident-related injuries, e.g., [5–10]. According to Nutley et al. [11], hoarding is associated with a clear disability burden, higher than that of major medical/psychiatric disorders such as diabetes, major depression, and chronic pain.

Current treatment options for this complex condition are limited, with the standard therapy being Cognitive Behavioural Therapy (CBT), based around the cognitive behavioural model of hoarding initially proposed by Frost and Hartl [12]. However, drop-out rates are high, and less than a third of individuals completing treatment programmes report clinically significant improvements [13, 14].

A key problem is that of comorbidity, as the symptoms of HD show considerable overlap with other psychiatric and neurodiverse conditions, and mood and anxiety disorders [15, 16], including OCD [17], ADHD [18, 19], depression [20, 21] and anxiety [11, 22]. Of particular relevance to this current research are studies which show intriguing links between HD and eating disorders (ED). Novara and colleagues [16], for example, measured the prevalence of hoarding symptoms in a sample of 124 ED patients with bulimia and binge eating, and found that over 20% of the ED sample exceeded the clinical cut-off point for hoarding severity, with particularly high scores in the *excessive acquisition* and *difficulty discarding* subscales of the *Savings Inventory Revised* (SI-R). Furthermore, it is evident that the two disorders display similar comorbidities, with elevated rates of depression and anxiety, e.g. [23, 24].

Of interest is the observation that people who hoard tend to have a higher BMI than non-hoarding controls [10, 25], suggesting a specific link between hoarding and difficulties with eating regulation. Raines and colleagues [26] confirmed a relationship between hoarding and higher BMI, and reported positive associations between binge eating (as measured by the *Eating Disorder Examination Questionnaire*) and hoarding severity (especially the *excessive acquisition* subscale of the SI-R). A further innovative study by Norberg and colleagues [27] found that in two groups with similar levels of BMI, participants with hoarding-related behaviours reported more problematic eating beliefs than the control group. Network analyses also confirm a link between hoarding and eating disorder symptoms [28, 29]. Another transdiagnostic factor which may explain the link between HD and ED is perfectionism. A number of studies have found increased levels of perfectionism in ED samples, e.g. [30, 31]. and it has been suggested that perfectionism is also elevated in HD [12, 32] with others finding that perfectionism was significantly associated with hoarding status [25]. Frost and colleagues [33] found elevated levels of perfectionism in people reporting currently high levels of acquisition, an association confirmed by [30]. Recently, Zaremohzzabieh et al. [34] found that maladaptive perfectionism moderated the relationship between emotional attachment to objects and digital hoarding and McCabe-Bennet et al. found that hoarding-related symptoms were associated with increased indecisiveness and some aspects of perfectionism [35]. A possible contributing role of perfectionism is also supported by the finding that higher levels of perfectionism are associated with poorer outcomes in CBT for HD [36]. However, there is relatively little current research into the relationship between perfectionism and HD, and the possibility that aspects of perfectionism might contribute to both HD and ED, specifically when the influence of mood is controlled for.

Hence, the aim of this study was to explore possible associations between hoarding severity and disordered eating, and the extent to which both disorders are associated with perfectionism. As previous research has revealed significant gender differences in eating disorder pathology, e.g. [37]. and in anxiety/depression [38], we controlled for gender and mood in the analysis. Our exploratory research questions are thus: :

1. The possible relationships between hoarding behaviours and severity, and various aspects of disordered eating.
2. The extent to which hoarding behaviours and severity, and aspects of disordered eating are independently associated with perfectionism.

## Method

### Design

The study employed a correlational design, to investigate the possible associations between hoarding, disordered eating symptoms and perfectionism, in a non-clinical sample, after controlling for gender, anxiety and depression.

### Participants

Due to the nature of the current study, it was impractical to conduct a power analysis to compute the sample size needed. Power analysis requires specific information, such as effect sizes, variances, and desired power levels. In cases where this information is not available or is difficult to estimate (e.g., in exploratory studies or novel areas of research), researchers may rely on past studies with similar designs or objectives to justify their sample size or generally accepted requirements for statistical procedures. Generally speaking, correlational research requires larger sample sizes for data interpretations, with sample sizes less than 200 rarely reported. Furthermore, on the back of evidence that online research has large attrition rates (see e.g. [26]. it was decided to aim for a large sample size of more than 200 participants to manage the expected drop-out rate and some participants not meeting the inclusion criteria.

Our initial sample consisted of 332 participants, recruited via opportunity sampling. Recruitment took place online (SurveyCircle and dedicated forums for sharing recruitment advertisements) and through word of mouth dissemination around university and research connections. Over-recruitment as compared to the required sample size was done to account for inevitable participant drop out due to incomplete responses and ineligibility for participation that are common in online research. Indeed, due to participant drop out and failure to meet inclusion criteria, the final sample consisted of 252 participants aged between 18 and 70 (M = 31.22; SD = 12.06), including 187 females (M = 29.95; SD = 11.55, range = 18–68) and 65 males (M = 31.22; SD = 11.44, range = 19–70).

### Materials

We used standardised validated psychometric measures, and referred to the published scoring information to compute total scores and subscales in each instance.

#### The Savings Inventory-Revised (SI-R)

The SI-R [39] comprises 23-items to assess the key hoarding behaviours of ‘*clutter*’, ‘*excessive acquisition*’, and ‘*difficulty discarding*’, and is often used as a screening tool for diagnosing HD. This psychometric measure specifies a symptom timeframe of the last week at the time of measurement. The possible score range is 0 to 92, with higher scores indicating greater hoarding severity. It has been shown to be an appropriate instrument for assessing hoarding behaviours in both clinical and non-clinical samples [39] and has been praised for its high internal reliability when used with control and clinical samples, with alpha = 0.84 and 0.94 respectively [40]. In this study, Cronbach’s alpha was 0.929 for the total score, 0.916 for *clutter*, 0.872 for *difficulty discarding*, and 0.785 for *excessive acquisition* subscales.

####  Eating Disorder Examination Questionnaire (EDEQ)

The EDEQ [41] is a 28-item self-rating-based assessment of eating disorder pathology and symptoms, measuring: ‘*eating restraint’* (5 items), ‘*eating concern*’ (5 items), ‘*body weight concern*’ (5 items), and ‘*body shape concern*’ (8 items) [42]. Items 13–18 use frequencies of incident which can be used to establish whether an eating disorder might be present and can also describe symptom frequency and/or severity. This psychometric measure specifies a symptom timeframe of the last 28 days at the time of measurement. In this study, we excluded these descriptive items (13–18), as these were not relevant to the study’s aim. The questionnaire has consistently demonstrated reliability across multiple studies as well as acceptable internal consistency [43]. In this study, Cronbach’s alpha was 0.954 for the total score, 0.854 for *eating restraint*, 0.850 for *eating concern*, 0.920 for *shape concern*, and 0.865 for *weight concern* subscales.

#### Bulimic Investigatory Test, Edinburgh (BITE)

The BITE [43] is a 33-item psychometric measure of bulimia symptoms and a reliable and valid measure, suitable for establishing bulimic disordered eating in both clinical and subclinical samples [43, 44]. This psychometric measure does not specify a symptom timeframe. It includes two subscales –‘*severity*’ comprising 3 items, with a total possible score of 39, and ‘*symptoms*’ comprising 30 items with a total possible score of 30. Cronbach’s alpha of this measure is α = 0.92 [44]. In this study, Cronbach’s alpha was 0.916 for ‘*severity*’ and 0.811 for the ‘*symptoms*’ subscales.

####  Frost Multidimensional Perfectionism Scale (FMPS)

The FMPS [45] is a validated and widely used measure of perfectionism. The original version is a 35-item psychometric measure assessing 6 facets of perfectionism. We used a revised version of this scale [46], with 4 facets being measured: ‘*personal standards*’, ‘*organization*’, ‘*parental expectations*’ and ‘*concern over mistakes*’. This measure does not specify a symptom timeframe. A total score can be produced by summing the subscales, excluding ‘*organization*’ as per published instructions [46], with the range of total scores being 29–145. The subscales are produced by summing up the respective items. Reliability of this measure has been reported to range between α = 0.71 and α = 0.86 [47]. In this study, Cronbach’s alpha was .930 for the total score, .924 for *concern over mistakes*.891 for *parents’ expectations*, .841 for *personal standards*, and .912 for *organization* subscales.

#### The generalised anxiety disorder questionnaire (GAD-7)

The GAD-7 [48] is a 7-item questionnaire measure of generalised anxiety, with a higher score indicating higher levels of anxiety. This measure specifies a symptom timeframe of the last 7 days at the time of measurement. It has strong internal reliability, with a Cronbach’s alpha of 0.89 [49] and strong validity and reliability as a measure of anxiety in the general population [50]. In this study, Cronbach’s alpha was 0.915.

#### The Patient Health Questionnaire (PHQ-9)

The PHQ-9 [51] is a 9-item questionnaire assessing depression, with a higher score indicating increased depressive symptoms. This psychometric measure specifies a symptom timeframe of the last 7 days at the time of measurement. It was selected because it is a quick and reliable measure of depression in a non-clinical sample and has high internal reliability, alpha = 0.85; [52]. In this study, Cronbach’s alpha was 0.865.

### Procedure

Data was collected via the online survey platform Qualtrics. Anonymity of participants was ensured by asking them to provide a codeword as an identifier. No compensation or incentives were offered. The survey included the participant information sheet, consent form, participant demographic questions, psychometric measures, and a debrief sheet. The psychometric measures were presented in the following order: PHQ-9, GAD-7, SI-R, FMPS, EDE-Q, and BITE. Time taken to complete the study was approximately 30 min. The data collection period commenced on February 28th 2023 and ended on June 1st 2023.

### Statistical approach

All analyses were conducted in JASP 0.17.2.1, using the Descriptive Statistics, Correlation, and Structural Equation Modelling (SEM) functions. The raw data is available at the Open Science Framework: https://osf.io/a8vhu/ and all data/analyses can be available by contacting the corresponding author.

### Frequentist statistical approach

The data were checked for outliers and total scores were computed for all questionnaires. When deciding how to handle outliers, the authors chose to abide by confidence intervals, consistent with the common approach to clinical data, where an outlier would be defined as being outside the 95% confidence level as determined by the current study sample size, sample mean and sample standard deviation, using a formula of $\:x\pm\:Z*\frac{s}{\surd\:n}$. We wanted to include extreme values where possible to increase the range of data points, and thus removed an outlier only if it was consistently outside the confidence intervals on more than two variables. This is to capture the true range of the subclinical population. Based on this, we removed one participant who did not fulfil the aforementioned criteria for retention. Normality testing of the data was conducted by employing the Shapiro-Wilk test. The results of this test indicated that none of the variables utilised in our main analyses were normally distributed (statistics available on request).

As Pearson’s correlation is a parametric test, which typically requires normal distribution of data, we chose to transform the variables using natural logarithmic transformation, which is a popular method for managing skewed data [53]. The current study used continuous variables. Whilst initial data inspection suggested deviations from normality, parametric tests are generally robust to moderate violations, particularly with our sample size. To further ensure the validity of analyses used, logarithmic transformations were applied, which improved distributional properties and allowed us to preserve the advantages of parametric methods (e.g., greater statistical power, the ability to estimate means and standard deviations directly, and the possibility of conducting follow-up or multivariate analyses).

Although non-parametric tests could have been applied, these are typically more appropriate for ordinal-level data or for cases with severe assumption violations and very small samples. Moreover, non-parametric methods test hypotheses about ranks rather than means which would not have aligned with the study’s research aims. For these reasons, it was determined as more appropriate to use transformed data with parametric analyses rather than switching to non-parametric tests.

The next step in the analysis was to check for significant associations between hoarding behaviours (as measured by SI-R), eating disorder behaviours (as measured by EDEQ & BITE), perfectionism (as measured by FMPS), after controlling for gender and depression (as measured by PHQ-9), and anxiety (as measured by GAD-7). As most of these scales also have subscales measuring different aspects of each construct, we included total scores and subscale scores in the correlational analysis. This allowed a more in-depth analysis of the relationship between the components of constructs. In total, 16 variables were included (SI-R clutter, SI-R discarding difficulties, SI-R acquisition difficulties, SI-R Total score, FMPS concerns over mistakes, FMPS concerns over parents’ expectations, FMPS personal standards, FMPS organization, FMPS total, EDEQ weight concern, EDEQ shape concern, EDEQ eating concern, EDEQ restraint, EDEQ total, BITE severity, and BITE symptoms).

We also conducted a t-test comparing male and female scores on all variables due to previous literature finding significant differences between males and females, particularly on eating pathology subscales [37]. For the t-test and the bivariate correlations, we used transformed scores using the natural logarithm of the values, hence, values shown for the aforementioned tests are transformed. However, for better understanding of the sample’s descriptive values, such as means and standard deviations, we used the original values in the descriptive statistics table.

Lastly, we conducted a mediation analysis to explore the possible mediation effect of the *organisation* subscale of the FMPS on the relationship between hoarding behaviour (SI-R) and eating pathology (BITE severity).

## Results

Initially, descriptive statistics, including mean and standard deviation were calculated for all variables, with questionnaire subscales considered as separate variables. These statistics can be found in Table 1. Any significant differences between the means for the two genders are reported on the last column of the table. 

**Table 1:** Descriptive statistics for all variables

Descriptive Statistics						
Variable	Gender	*N*	*M*	*SD*	*t*	*P*
Age	Male	65	31.215	11.437		
	Female	187	29.947	11.549		
	Both	252	30.274	11.511	0.765	0.445
PHQ9 Total	Male	65	6.200	4.634		
	Female	187	9.342	5.856		
	Both	252	8.532	5.726	-3.551	< 0.001
GAD7 Total	Male	65	5.738	5.197		
	Female	187	8.134	5.720		
	Both	252	7.516	5.678	-2.847	0.005
SI-R Total	Male	65	20.062	12.592		
	Female	184	23.614	14.101		
	Both	249	22.687	13.787	-1.935	0.054
SI-R Clutter	Male	65	5.877	5.592		
	Female	186	7.199	6.458		
	Both	251	6.857	6.261	-0.654	0.514
SI-R Discarding	Male	65	7.631	5.270		
	Female	187	8.717	5.520		
	Both	252	8.437	5.467	-0.745	0.457
SI-R Acquisition	Male	65	6.554	4.294		
	Female	185	7.832	4.513		
	Both	250	7.500	4.484	-2.126	0.035
FMPS Mistakes	Male	65	35.231	10.025		
	Female	187	41.080	10.902		
	Both	252	39.571	10.967	-3.714	< 0.001
FMPS Parents Expectations	Male	65	22.877	6.163		
	Female	187	24.652	8.311		
	Both	252	24.194	7.840	-1.075	0.283
FMPS Standards	Male	65	23.215	5.146		
	Female	187	24.214	5.284		
	Both	252	23.956	5.257	-1.220	0.224
FMPS Organization	Male	65	21.615	5.595		
	Female	187	22.374	4.919		
	Both	252	22.179	5.101	-1.242	0.215
FMPS Total	Male	65	81.323	17.203		
	Female	187	89.947	20.136		
	Both	252	87.722	19.754	-2.937	0.004
EDEQ Restraint	Male	65	1.246	1.501		
	Female	187	1.953	1.643		
	Both	252	1.771	1.634	-2.259	0.025
EDEQ Eating Concern	Male	65	0.698	0.955		
	Female	187	1.600	1.529		
	Both	252	1.367	1.457	-3.830	< 0.001
EDEQ Shape Concern	Male	65	1.858	1.561		
	Female	187	3.127	1.784		
	Both	252	2.800	1.813	-3.784	< 0.001
EDEQ Weight Concern	Male	65	1.535	1.395		
	Female	187	2.876	1.701		
	Both	252	2.530	1.728	-4.527	< 0.001
EDEQ Total	Male	65	1.334	1.151		
	Female	187	2.389	1.470		
	Both	252	2.117	1.467	-4.851	< 0.001
BITE Symptoms	Male	53	8.698	7.397		
	Female	143	13.972	7.360		
	Both	196	12.546	7.717	-5.516	< 0.001
BITE Severity	Male	19	11.211	6.754		
	Female	57	10.263	4.623		
	Both	76	10.500	5.204	0.315	0.754

The *p*-values and t-values are depicted using transformed scores. The number (N), means (M), and standard deviations (SD) have been presented using non-transformed values for better understanding of the sample

*PHQ-9*  Patient Health Questionnaire–9, *GAD-7*  Generalized Anxiety Disorder–7, *SI-R*  Saving Inventory–Revised (Clutter, Discarding, Acquisition subscales), *FMPS*  Frost Multidimensional Perfectionism Scale (Mistakes, Parental Expectations, Standards, Organization subscales), *EDE-Q*  Eating Disorder Examination–Questionnaire (Restraint, Eating Concern, Shape Concern, Weight Concern subscales), *BITE*  Bulimic Investigatory Test, Edinburgh (Symptoms, Severity subscales)

Overall, there were numerous differences between the means for the two genders. Females scored significantly higher on all subscales of eating pathology, bulimic inventory scale, mood measures and perfectionism total score. However, the self-perceived severity of bulimia nervosa symptoms, as measured by the BITE *severity* subscale, was not significantly different between the genders. Neither were there significant gender differences in hoarding measures other than the excessive *acquisition* subscale, where females scored significantly higher than the males. There were no significant differences between males and females on any of the perfectionism subscale measures. Regarding the variance in sample sizes across the psychometrics used in this study, we noticed that the BITE *severity* subscale had significantly fewer participants, compared to the rest of the psychometric measures. This is because the aforementioned subscale is computed based on three items which use a Likert-type scale to rate the frequency of binge eating and compensatory behaviours, however remaining items are rated using a dichotomous scale (‘yes’ and ‘no’), As this study recruited participants from the general population, it is possible that for a significant portion of our sample, the frequency of binge eating and compensatory behaviours did not apply.

Where there was not a full sample of participants available due to missing data, we used a reduced number of participants for analyses purposes. See Table 1 for sample sizes associated with each variable.

### Bivariate analyses

**Fig. 1:**
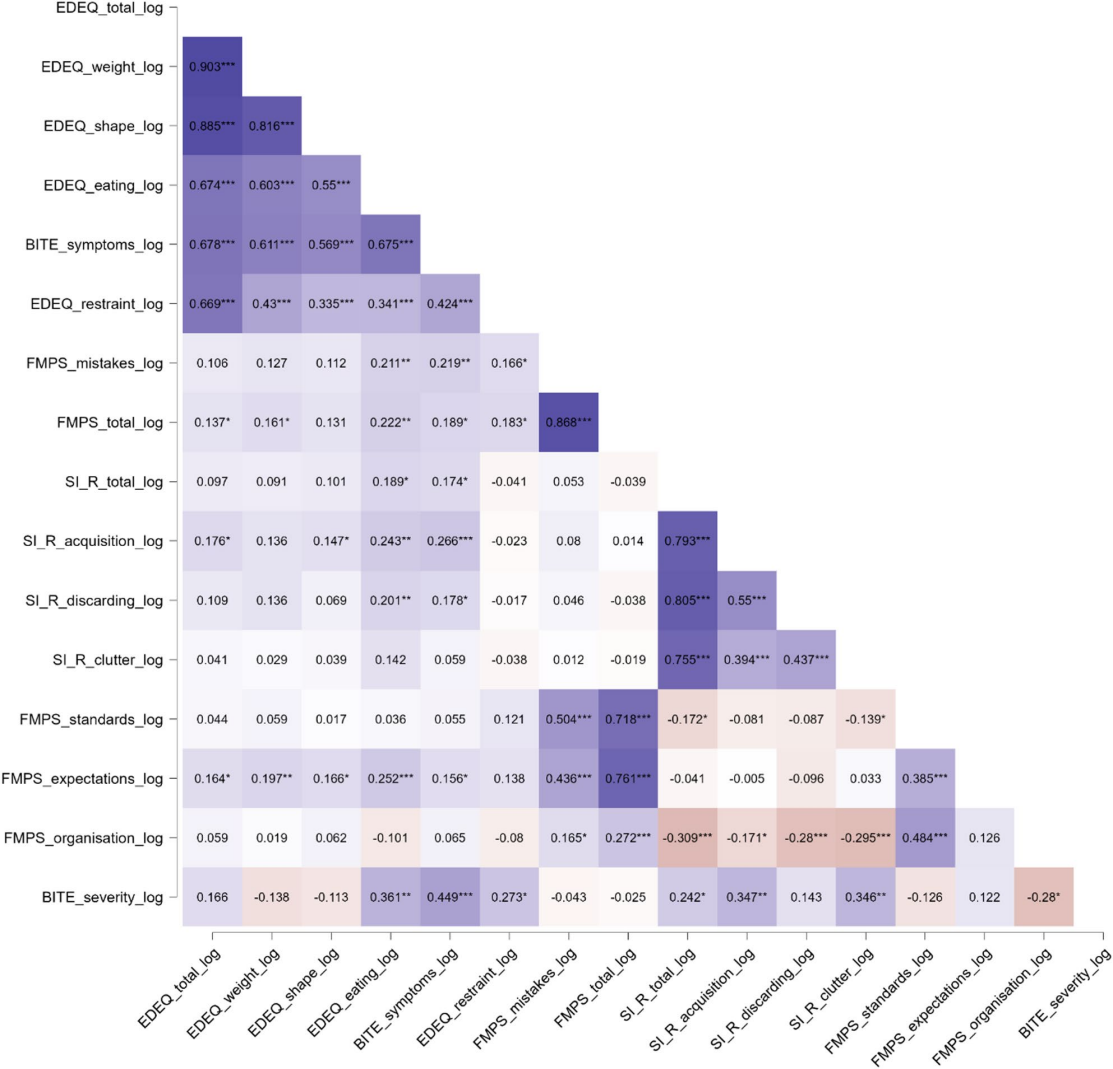
Pearson’s r heatmap with correlations controlled for gender and mood variables (anxiety and depression). *N* = 251. Note. PHQ-9 = Patient Health Questionnaire–9; GAD-7 = Generalized Anxiety Disorder–7; SI-R = Saving Inventory–Revised (Clutter, Discarding, Acquisition subscales); FMPS = Frost Multidimensional Perfectionism Scale (Mistakes, Parental Expectations, Standards, Organization subscales); EDE-Q = Eating Disorder Examination–Questionnaire (Restraint, Eating Concern, Shape Concern, Weight Concern subscales); BITE = Bulimic Investigatory Test, Edinburgh (Symptoms, Severity subscales); log = logarithmic transformation

Due to the number of statistical differences between mean scores for males and females and strong correlations between the variables of interest and the mood variables, we ran a partial correlation, assessing possible relationships between hoarding severity, disordered eating and perfectionism, controlling for gender, anxiety and depression, see Fig. 1. The range of r values from the initial correlation was between r 0.926 and *r* − .261.

After controlling for gender and mood, hoarding showed significant associations with both disordered eating and perfectionism. In relation to eating pathology, the SI-R total score was unrelated to most EDE-Q subscales, with the exception of a positive correlation with *eating*.

*concern* but it was positively associated with both BITE *symptoms* and *severity*. Within the SI-R subscales, e*xcessiv*e *acquisition* and *clutter* were positively correlated with BITE *severity*, and *excessive acquisition* emerged as the most notable, showing additional positive associations with EDE-Q *eating concern*, *shape concern*, and *total*, as well as BITE *symptoms* and *severity*.

When considering hoarding and perfectionism, the only significant links were negative, with all SI-R scores (*total*, *excessive acquisition*, *difficulty discarding*, and *clutter*) inversely correlated with FMPS *organization*.

In contrast, associations between eating pathology and perfectionism were largely positive. EDE-Q *total* and *weight concern* were positively related to FMPS *parental expectations* and *total*, while EDE-Q *shape concern* was associated with *parental expectations*, and EDE-Q *eating concern* with *parental expectations*, *total*, and *concern* over *mistakes*. Similarly, BITE *symptoms* were positively correlated with FMPS *parental expectations*, *concern* over *mistakes*, and *total*. The only negative association in this domain was between BITE *severity* and FMPS *organization*.

Taken together, these findings indicate that key aspects of hoarding were positively associated with several aspects of disordered eating, most consistently through the *excessive acquisition* subscale. Eating pathology, as expected, was positively associated with perfectionism. However, hoarding and perfectionism were linked only through a negative correlation with *organization*.

### Mediation analysis

**Fig. 2:**
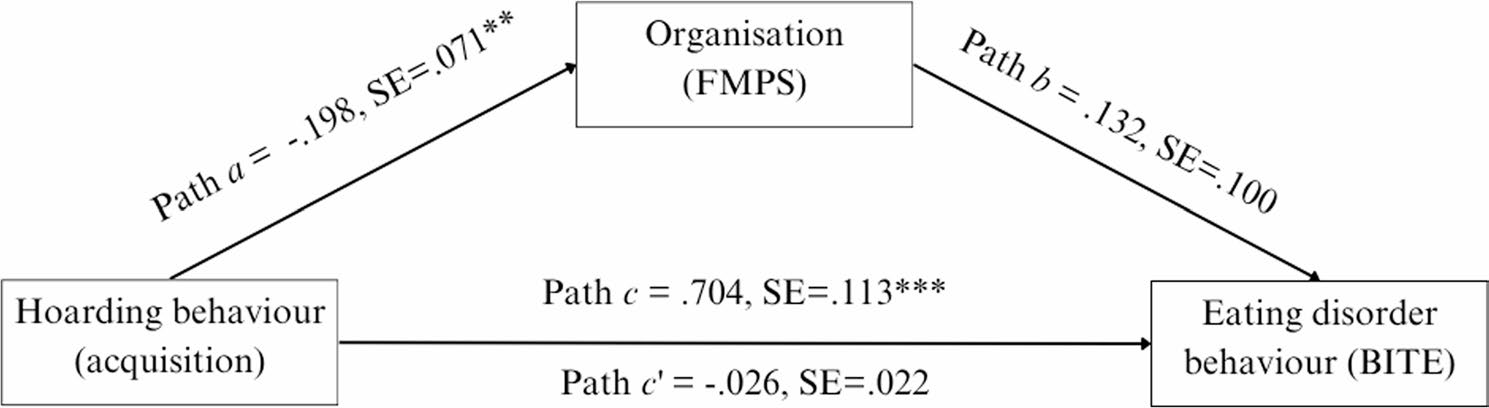
Mediation analysis. Significant indirect pathway from hoarding behaviours to eating disorder behaviours measured by BITE severity subscale and mediated by FMPS organisation subscale. Note. Standardised coefficients are reported. c': indirect effect *p <. 05, ** *p* <. 01, *** *p* <. 001

The previous analyses revealed significant associations between excessive acquisition and eating disorders, including the perfectionism organisation subscale, so a mediation analysis (with no additional covariates) was conducted to explore whether organisation difficulties mediate the relationship between excessive acquisition and eating disorder symptoms (Fig. 2). It is appropriate to use mediation analysis for exploratory purposes [54]. The analysis revealed that there is a significant direct effect (Path *c)*, which suggests that the relationship between excessive acquisition and eating behaviours is primarily direct, and is not mediated through organisation difficulties. The lack of significance in the indirect effect (Path *c’*) implies that the mediation hypothesis (where organisation difficulties would explain the relationship between acquisition and eating behaviours) is not supported in this case. Path *a* is significant and suggests that acquisition does affect organisation difficulties, but Path *b* is not significant, showing that organisation difficulties do not significantly influence eating behaviours in the presence of excessive acquisition.

In short, this mediation analysis rules out a mediation pathway that some might assume exists, showing that although perfectionism correlates with both hoarding and disordered eating symptoms, the link is not explained by this organisational aspect. *Excessive acquisition* predicts disordered eating symptoms directly, independent of organisation-related perfectionism.

## Discussion

Hoarding Disorder (HD) and Eating Disorders (ED) share some comorbidities, with both for example showing higher rates of depression and anxiety, e.g. [55, 56]. Studies show that ED samples display a high prevalence of hoarding behaviours, e.g. [16], with particularly high scores being seen on the *excessive acquisition* and *difficulty discarding* subscales of the SI-R in eating disordered participants. As people who hoard are known to display higher BMI than non-hoarding controls [10, 25], it has been suggested that they have difficulties with eating regulation. One potential transdiagnostic factor is that of perfectionism, with elevated levels being reported in ED samples, e.g. [28]. and HD samples [57].

In this exploratory study we examined the extent to which components of hoarding behaviours, and hoarding severity were associated with aspects of disordered eating; and the extent to which hoarding and disordered eating were associated with perfectionism.

We initially found significant gender differences on measures of disordered eating, anxiety and depression, and total score for perfectionism (in each case females scoring significantly higher than males). The females in our sample also scored significantly higher than males on the ‘acquisition’ subscale of the hoarding measure, with this being unanticipated. We therefore controlled for gender and mood in our subsequent analyses.

Research question 1 was concerned with the possible relationships between hoarding behaviours and severity, and aspects of disordered eating. We indeed found significant associations between hoarding-related behaviours and disordered eating. There were weak associations with eating disorder symptoms in general, as measured by the EDE-Q subscales, but stronger associations with bulimic symptom *severity*, as measured by the BITE. Further investigation into the subcomponents of hoarding revealed that the associations were strongest for the *excessive acquisition* aspect of hoarding, which correlated positively with EDEQ *eating concern*, *shape concern* and total scores and with the BITE measures of bulimic *symptoms* and *severity*. The subsequent mediation analysis further revealed a direct relationship between excessive acquisition and disordered eating.

These data do suggest that hoarding-related behaviour and disordered eating may share some commonalities, and we speculate that these may include a dysfunction in behavioural regulation which drives the excessive acquisition in hoarding and certain aspects of disordered eating, notably binge-eating which may indeed reflect a general deficit in impulse-control [58]. Hoarding has been linked with binge eating in previous research, e.g. [59]. with he dimension of hoarding most associated with binge eating being ‘acquisition’ [16]; and de Mattos et al. [60] speculated that the psychological need to excessively gather and store items may be a shared process that underpins disordered eating and the compulsive acquisition of items.

However, while it is notable that bulimic ‘severity’ was associated with hoarding-related behaviour and ‘acquisition’, the BITE questionnaire used in this study was designed to detect all symptoms of bulimia nervosa, and does not specifically measure binge eating, so our suggestion that the link between eating disorders and hoarding is primarily due to a link between binge eating and hoarding remains to be confirmed with the use of a specific measure of binge eating.

Our second research question considered the idea that possible connections between hoarding-related behaviours and eating disorders might be explained by the shared transdiagnostic factor of perfectionism, which previous studies have identified in relation to both disorders [12, 25, 30–32]. We found the expected associations with disordered eating behaviours, particularly with regard to the c*oncern over mistakes* subscale of the perfectionism measure. No overall association between hoarding-related behaviours and perfectionism was found, although there was an interesting negative correlation between the *organization* subscale of perfectionism measure and hoarding-related behaviours (total score and all subscale scores). This is consistent with McCabe-Bennet and colleagues [35] who found the same association between hoarding-related behaviours and the *organization* subscale. They also found an association between hoarding and the *doubts about action* subscale of the FMPS but we were unable to confirm this as this subscale is excluded from the revised version of the FMPS used in our study.

This consistent negative association between the *organization* subscale of perfectionism and all hoarding subscales in our study is interesting. This subscale specifically measures the extent to which people desire to be, and see themselves as, ‘neat’ and ‘organised’ It therefore makes intuitive sense that high scores on measures of hoarding would be associated with low scores on this subscale. This finding suggests that a further transdiagnostic link between binge-eating and hoarding disorder may be poor executive function, which has been found previously to be associated both with hoarding disorder [19] and with binge eating disorder [61]. We did not collect any data relating to executive function, and so future research should consider this as a potential factor.

Interestingly, the mediation analysis also indicated that the relationship between excessive acquisition and eating behaviours is primarily direct, with organisation difficulties not serving as a significant mediator. This suggests that while hoarding behaviours are associated with both eating behaviours and organisational difficulties, the pathway from hoarding to eating behaviours is not explained by this dimension of perfectionism. This highlights the need to explore alternative mechanisms or other dimensions of perfectionism. It also adds nuance to theory: perfectionism is multidimensional, and not all subcomponents play the same role. Showing that organisation difficulties do *not* mediate highlights the need to examine other perfectionism dimensions.

The excessive acquisition in hoarding and binge eating might also reflect a dysfunction in emotion regulation - the ability to understand and differentiate emotions, and problems in modulating emotions. As emotion dysregulation might exacerbate negative emotional states by leading to increased arousal, distress and avoidance, it is thought to contribute to several psychiatric disorders, e.g. [62]. Increased emotional dysregulation has been found to predict hoarding severity in a non-clinical sample, e.g. [63]. and difficulties in emotion regulation mediated an association between hoarding severity (especially ‘acquisition’) and binge eating [26]. Future research should address this possible link.

Another transdiagnostic factor which we did not measure, and which may be important is that of intolerance of uncertainty (IoU) – the perceived inability to endure an emotional response triggered by a lack of certain information, and maintained by the subsequent perceived uncertainty [64]. Several studies have noted that IoU is a significant predictor of hoarding behaviours, with particularly close associations with *acquisition* and *difficulty discarding* [65]. IoU is also found to be higher in individuals with eating disorders and may represent an important vulnerability and maintenance factor [66].

We acknowledge that this research is limited by the use of a cross-sectional convenience sample, drawn from a non-clinical population, which contained a predominance of female participants. We also acknowledge that the reliance on self-report measures may introduce biases (e.g. social desirability) and problems in recall, and may reflect the current emotional state of the participant. Furthermore, whilst this study suggests an important avenue to be explored to inform clinical practice, more substantial evidence is required to make more generalizable interpretations concerningpatient samples.

It is possible that a different (i.e. clinical) sample might reveal different associations, although we suspect that these might in fact be strengthened, a possibility corroborated by the lack of extreme scores in our sample.

Furthermore, this research applied mediation model to explore the links between variables. Whilst a mediation model was considered most appropriate given the lack of existing knowledge and the available prior literature, it may be that a moderation framework would have revealed more substantial effects. This was not explored due to the lack of theoretical justification for this approach.

In conclusion, we found a significant association between aspects of disordered eating and hoarding-related behaviours, particularly excessive acquisition. Initial predictions of a positive association between hoarding-related behaviours and perfectionism were not confirmed after controlling for gender and mood, and we found only a negative association between hoarding-related behaviours and ‘organization’. Finally, the predicted association between aspects of disordered eating and perfectionism persisted at all levels of analysis.

In light of our preliminary findings, it is possible that binge eating and excessive acquisition (key components of the two disorders) are associated, rather than the disorders as a whole. This needs further investigation as our significant findings relate to the BITE severity subscale, which includes three items assessing binge eating, compensatory behaviors, and fasting. This subscale reflects symptom intensity among a subset of participants, not the presence of symptoms across the full sample. Our findings do not support the hypothesis that any aspect of perfectionism is a transdiagnostic factor linking disordered eating and hoarding-related behaviours.

## Data Availability

The data is available at the Open Science Framework at [https://osf.io/a8vhu/] (https://eur02.safelinks.protection.outlook.com/?url=https%3A%2F%2Fosf.io%2Fa8vhu%2F&data=05%7C02%7Cnick.neave%40northumbria.ac.uk%7Ce9a086d2ef524ea0af5108dc471df13b%7Ce757cfdd1f354457af8f7c9c6b1437e3%7C0%7C0%7C638463441121027202%7CUnknown%7CTWFpbGZsb3d8eyJWIjoiMC4wLjAwMDAiLCJQIjoiV2luMzIiLCJBTiI6Ik1haWwiLCJXVCI6Mn0%3D%7C0%7C%7C%7C&sdata=Br89hLH1ryl6ZJN1gOCBRBdYzhkPhDK9vM1C3ZYXl9U%3D&reserved=0).

## Abbreviations

ADHD: Attention-Deficit/Hyperactivity Disorder
BITE: Bulimic Investigatory Test, Edinburgh
CBT: Cognitive Behavioural Therapy
ED: Eating Disorders
EDE-Q: Eating Disorder Examination–Questionnaire
FMPS: Frost Multidimensional Perfectionism Scale
GAD-7: Generalized Anxiety Disorder–7
HD: Hoarding Disorder
M: Mean
N: Number of participants
OCD: Obsessive-Compulsive Disorder
PHQ-9: Patient Health Questionnaire–9
SD: Standard Deviation
SEM: Structural Equation Modelling
SI-R: Saving Inventory–Revised
